# Oral beclomethasone dipropionate is an effective treatment for immune checkpoint inhibitor induced colitis

**DOI:** 10.1136/jitc-2022-005490

**Published:** 2022-09-16

**Authors:** James L Alexander, Hajir Ibraheim, Camellia Richards, Ben Shum, Polychronis Pavlidis, Nikki Hunter, Julian P Teare, Andrew Wotherspoon, Andrew Furness, Samra Turajlic, Lisa Pickering, James Larkin, Ally Speight, Sophie Papa, Nick Powell

**Affiliations:** 1 Department of Gastroenterology, Royal Marsden NHS Foundation Trust, London, UK; 2 Department of Metabolism, Digestion and Reproduction, Imperial College London, London, UK; 3 Renal & Skin Units, Royal Marsden NHS Foundation Trust, London, UK; 4 Experimental Immunobiology, King's College London, London, UK; 5 Department of Gastroenterology, King's College Hospital NHS Foundation Trust, London, UK; 6 Department of Histopathology, Royal Marsden NHS Foundation Trust, London, UK; 7 Cancer Dynamics Laboratory, The Francis Crick Institute, London, UK; 8 Department of Gastroenterology, Newcastle Upon Tyne Hospitals NHS Foundation Trust, Newcastle Upon Tyne, UK; 9 School of Cancer and Pharmaceutical Sciences, King's College London, London, UK

**Keywords:** Inflammation, Immunotherapy

## Abstract

**Introduction:**

Systemic corticosteroids are the mainstay of treatment for immune checkpoint inhibitor induced (CPI) colitis but are associated with complications including life-threatening infection. The topically acting oral corticosteroid beclomethasone dipropionate (BD) is an effective treatment for mild to moderate flares of ulcerative colitis, and has fewer side effects than systemic corticosteroids. We hypothesized that BD would be an effective treatment for CPI-induced colitis.

**Methods:**

We performed a retrospective analysis of all patients who started BD for CPI-induced colitis at three UK cancer centers between November 2017 and October 2020. All patients underwent endoscopic assessment and biopsy. The initial regimen of BD was 5 mg once daily for 28 days. Data were collected from electronic patient records. Clinical outcomes were assessed at 28 days after initiation of treatment.

**Results:**

Twenty-two patients (14 male) with a median age of 64 (range 45–84) with CPI-induced colitis were treated with BD. At baseline, the median number of loose stools in a 24-hour period was six (common terminology criteria for adverse events, CTCAE grade diarrhea=2). Thirteen patients (59%) were dependent on systemic corticosteroids prior to starting BD. Baseline sigmoidoscopy showed moderate inflammation (Mayo Endoscopic Score (MES) = 2) in two patients (9%), mild inflammation (MES=1) in nine patients (41%) and normal findings (MES=0) in eleven patients (50%). Twenty patients (91%) had histopathological features of inflammation. All 22 patients (100%) had a clinical response to BD and 21 (95%) achieved clinical remission with a return to baseline stool frequency (CTCAE diarrhea=0). Ten patients (45%) had symptomatic relapse on cessation of BD, half within 7 days of stopping. All patients recaptured response on restarting BD. No adverse events were reported in patients treated with BD.

**Conclusions:**

Topical BD represents an appealing alternative option to systemic immunosuppressive treatments to treat colonic inflammation. In this study, BD was effective and safe at inducing remission in CPI-induced colitis, which was refractory to systemic corticosteroids. Further randomized studies are needed to confirm these findings and determine the optimum dosing regimen.

**WHAT IS ALREADY KNOWN ON THIS TOPIC**

- Checkpoint inhibitor (CPI)-induced colitis can affect up to 50% of patients taking CPI therapy, in many cases leading to cessation of this life-saving treatment.
- Systemic corticosteroids are the mainstay of treatment for CPI-induced colitis, but are associated with complications including life-threatening infection.
- The topically acting oral corticosteroid beclomethasone dipropionate (BD) is an effective treatment for mild to moderate flares of ulcerative colitis, and has fewer side effects than systemic corticosteroids.

**WHAT THIS STUDY ADDS**

- Twenty-one of 22 patients treated with BD in our study achieved clinical remission from CPI-induced colitis, and in 55% remission was durable.
- In those patients who had relapse of their diarrhea symptoms on cessation of BD, all recaptured response after restarting BD.
- There were no adverse events reported in patients on BD.

**HOW THIS STUDY MIGHT AFFECT RESEARCH, PRACTICE OR POLICY**

- This study suggests that BD is an effective treatment for CPI-induced colitis.
- Given its more favorable safety profile, topical BD represents an appealing alternative option to systemic immunosuppressive therapies in patients with mild or moderate CPI-induced colitis.
- Randomized trials are now needed to confirm these results and determine BD’s position in CPI-induced colitis treatment algorithms.

## Introduction

Immune checkpoint inhibitor (CPI) treatment has been a game-changer in the management of many advanced cancers. CPIs block the immune inhibitory molecules cytotoxic T-lymphocyte antigen-4 (CTLA-4), programmed death receptor-1 (PD-1), programmed death ligand 1 (PD-L1) and lymphocyte activation gene 3 (LAG-3), thus boosting the immune mediated response to cancer.^1–4^ Patients with advanced melanoma, previously a diagnosis associated with poor outcomes, now have median survival of more than 5 years.^5^ However, this increased immune activity comes at the cost of triggering off-target immune-mediated injury to other tissues and organs.^6^ Diarrhea and gastrointestinal (GI) tract inflammation affect up to 50% of patients taking immune CPI drugs,^7^ which can lead to hospitalization and bowel perforation in extreme cases. Unfortunately, the development of high-grade GI toxicity necessitates cessation of this life-saving anticancer therapy in most cases.

Guidelines recommend initial treatment of CPI-induced colitis with systemic corticosteroids.^8 9^ In moderate to severe cases, or cases refractory to oral prednisolone, high-dose intravenous steroid treatment is advised. High-dose corticosteroids are frequently administered for prolonged periods, but this strategy has several drawbacks. In addition to many well-documented side effects,^10^ including weight gain, reduced bone density and sleep disturbance, retrospective evidence points to steroid-related severe or life-threatening infection in patients with CPI-induced colitis.^11 12^ Moreover, some studies have suggested that high-dose steroid exposure may be associated with reduced overall survival in patients with cancer treated with CPI.^13 14^ A similar problem may be encountered with other systemic immunosuppressive treatments. Patients with cancer treated with biological agents, such as anti-TNF monoclonal antibodies may also be at risk of serious infection and reduced overall survival.^15^ In addition to safety concerns regarding systemic immunosuppression in CPI-induced colitis, the efficacy of current treatment options has also been challenged. A meta-analysis of 33 studies reporting outcomes of systemic corticosteroid treatment of CPI-induced colitis reported response rates of only 59%.^16^ Similar results were seen in infliximab treatment of corticostertoid refractory CPI-induced colitis, especially if robust outcome measures were adopted. For instance, in the largest study of infliximab treatment (127 patients with corticosteroid refractory CPI-induced colitis), only half of patients were in corticosteroid-free remission (common terminology criteria for adverse events (CTCAE) = 0) at 12 and 26 weeks after treatment.^17^ Accordingly, there is an unmet need for additional safe and effective anti-inflammatory therapies in CPI-induced colitis

An important first-line investigation for patients with suspected CPI-induced colitis is lower GI endoscopy. Common findings include erythema, erosions, ulceration and mucosal edema, with the presence of ulceration and extensive disease representing a more severe steroid refractory phenotype.^18 19^ However, in up to 37% of patients, endoscopic findings are normal, but histological analysis reveals inflammation—a phenotype referred to as ‘microscopic colitis’.^20–23^ In some cases, this phenotype appears to represent a distinct, less aggressive disease subtype, although in others, when endoscopic examination has been delayed, it may reflect the recent resolution of macroscopic changes secondary to immunosuppressive therapy. A variety of histological features have been observed including the typical histological findings conventionally observed in CPI-induced colitis, including acute and chronic inflammation, neutrophilic inflammation and crypt abscess formation,^24 25^ and the features of conventional/sporadic microscopic colitis, such as lymphocytic or collagenous colitis.^26^

Beclomethasone dipropionate (BD) is a second generation, controlled-release topical corticosteroid, which is metabolized in the gut mucosa to the active metabolite beclomethasone-17-monoproprionate. It is released in the distal small intestine and proximal colon to deliver therapy to the whole colon. Beclomethasone-17-monoproprionate has high bioavailability and undergoes extensive first pass metabolism in the liver, limiting its systemic bioavailability and reducing systemic side effects,^27^ which mitigates the requirement for prophylactic bone and stomach protection that is usually required with systemic corticosteroids. Randomized controlled trial evidence shows BD to be non-inferior in efficacy to oral prednisolone in the treatment of active mild to moderate ulcerative colitis.^28^ UK guidelines suggest BD as an alternative treatment to oral prednisolone in patients with mild to moderate ulcerative colitis in whom avoidance of systemic corticosteroids is desired.^29^

Given that there is considerable overlap in clinical, endoscopic and histological features between conventional inflammatory bowel disease, microscopic colitis and CPI-induced colitis, there is an attractive rationale for the use of topical corticosteroid treatment in the latter. Moreover, CPI-treated patients, in addition to having advanced malignancy, are often older and multimorbid, with higher rates of systemic corticosteroid related infections than patients with IBD. In this study, we sought to evaluate the safety and efficacy of BD in the treatment of CPI-induced colitis.

## Methods

A retrospective analysis was performed on all patients treated with BD for CPI-induced colitis at three cancer centers: The Royal Marsden Hospital, Guy’s and St Thomas’ National Health Service (NHS) Trust (both London, UK) and Newcastle upon Tyne Hospitals NHS Foundation Trust, Newcastle, UK, between November 2017 and October 2020.

The inclusion criteria were adult patients with any cancer who had a diagnosis of CPI-induced colitis (defined by presence of symptoms and the absence of a more likely diagnosis), which was treated with at least one course of BD. All patients had symptoms consistent with CPI-induced diarrhea or colitis according to the Common Terminology Criteria for Adverse Events (CTCAE) version 5.0. All patients had commenced ‘conventional’ therapy with systemic corticosteroids ± second-line immunosuppressive therapy (eg, infliximab and vedolizumab), and undergone a screen for alternative causes of symptoms such as GI infection. As a prerequisite for initiating BD, patients had either failed to achieve clinical remission (defined as a return to baseline stool frequency) or had experienced clinical relapse on stopping treatment with systemic corticosteroids ± other immunosuppressive therapy. Furthermore to be eligible for BD treatment, patients first needed to have endoscopically normal or mild to moderate colitic changes only (erythema, loss of vascular pattern and small erosions) on flexible sigmoidoscopy, with no evidence of extensive inflammation or colonic ulceration. Patients receiving BD for any other indication, such as conventional inflammatory bowel disease, were excluded.

Patients were treated with BD after agreement between their Gastroenterologist and their Oncologist. The dosing regimen was initially BD 5 mg daily for 28 days. In the event of relapse following cessation of BD, some patients were treated with extended courses at the discretion of their clinician.

Clinical data including patient demographics, symptoms, investigation results and treatments were retrieved from the hospital electronic patient records. CTCAE was used to determine the severity of diarrhea. Mayo endoscopy scores were assigned based on findings at index lower GI endoscopy. Clinical response to BD was defined as any improvement in GI symptoms. Clinical remission was defined as return to baseline stool frequency and, if present, resolution of abdominal pain and urgency. Durable remission was defined as the absence of GI symptoms 12 weeks following cessation of BD therapy. Spearman correlation of CTCAE grade and Mayo endoscopy score was performed. Data analysis was performed in Graphpad Prism V.9.0.0.

## Results

### Baseline characteristics

Twenty-two patients were treated with BD and included in the study. Baseline features are summarized in table 1. Combination anti-CTLA-4 and anti-PD-1 therapy was used in eight patients. Ten patients received anti-PD-1 monotherapy and one patient received combination anti-CTLA-4 and anti-LAG3 therapy. One patient was randomized to either anti-CTLA-4 monotherapy or anti-PD-1 monotherapy or combination anti-CTLA-4 and anti-PD-1 therapy. The median number of days between CPI treatment initiation and the onset of diarrhea was 103 (range 6–374). The majority of patients (73%) developed GI symptoms after five doses or fewer of CPI therapy, but two patients (9%) did so after more than 10 cycles of CPI. All twenty-two patients developed diarrhea. Other symptoms reported by patients included nocturnal defecation (n=7; 32%), fecal urgency (n=5; 23%), abdominal pain or cramps (n=6; 27%) and rectal bleeding (n=3; 14%). Nine patients (41%) were hospitalized due to their diarrhea or colitis symptoms. Of note, sixteen patients (73%) also developed one or more extraintestinal immune-related adverse event (table 1).

**Table 1:** Patient demographics

	No of patients
Male:female	14:8
Median age (range)	64 (45–84)
Type of cancer	
Melanoma	15 (68%)
Renal	4 (18%)
Urothelial	2 (9%)
NSCLC	1 (4%)
Charlson Comorbidity Index median	8 (range: 6–10)
Immunotherapy	
Anti-CTLA-4 and anti-PD-1	8 (36%)
Anti-PD-1 only	10 (45%)
Anti PD-1 and anti-LAG-3	1 (4%)
Anti-CTLA-4/anti-PD-1 monotherapy or combination	1 (4%)
No of immunotherapy cycles prior to diarrhea or colitis	
1–2	5 (23%)
3–5	11 (50%)
6–10	4 (18%)
>10	2 (9%)
Gastrointestinal symptoms	
Diarrhea	22 (100%)
Nocturnal defecation	7 (32%)
Urgency	5 (23%)
Abdominal pain/cramps	6 (27%)
Bleeding	3 (14%)
Other IrAEs	
Respiratory	3
Endocrine	6
Hepatological	2
Dermatological	8
Hematological	1
Rheumatological	1
Gastroenterological	2
Neurological	1
No IrAEs	6
Treatments for CPI colitis prior to BD	
Systemic corticosteroids	22 (100%)
Intravenous corticosteroids	11 (50%)
Anti-TNF (infliximab/adalimumab)	13 (59%)
Vedolizumab	3 (14%)
Adverse events on systemic corticosteroids	
Any adverse event	6 (27%)
Poor glycemic control	2 (9%)
Weight gain	1 (4%)
Infection	3 (14%)
Median no of days of continuous CS prior to BD (IQR)	58 (27–170)
Median no of days between last biological dose and BD initiation (range)	8 (1–190)

BD, beclomethasone dipropionate; CPI, checkpoint inhibitor.

Prior to BD treatment, all 22 patients had been treated with systemic corticosteroids. Eleven (50%) received intravenous corticosteroids (table 1). Thirteen patients (59%) were systemic corticosteroid dependent (defined as the requirement for prednisolone ≥10 mg per day, or equivalent, for at least 3 months, or relapse within 3 months of stopping systemic corticosteroid therapy) when starting BD. The median number of days of continuous corticosteroid therapy prior to starting BD was 58, and nine patients (41%) had been on systemic corticosteroids for over 130 days of the previous year. Six patients (27%) had adverse events attributable to systemic corticosteroid treatment, including poor glycemic control, weight gain and infection. Biologic therapy had also been administered in the majority: 13 patients (59%) had failed to achieve remission with infliximab (median number of doses=3 (range 1–4)), of which three patients (14%) subsequently received three doses (n=2) or five doses (n=1) of vedolizumab, with inadequate response.

### Clinical, endoscopic, and histological characterisation

**Figure 1:**
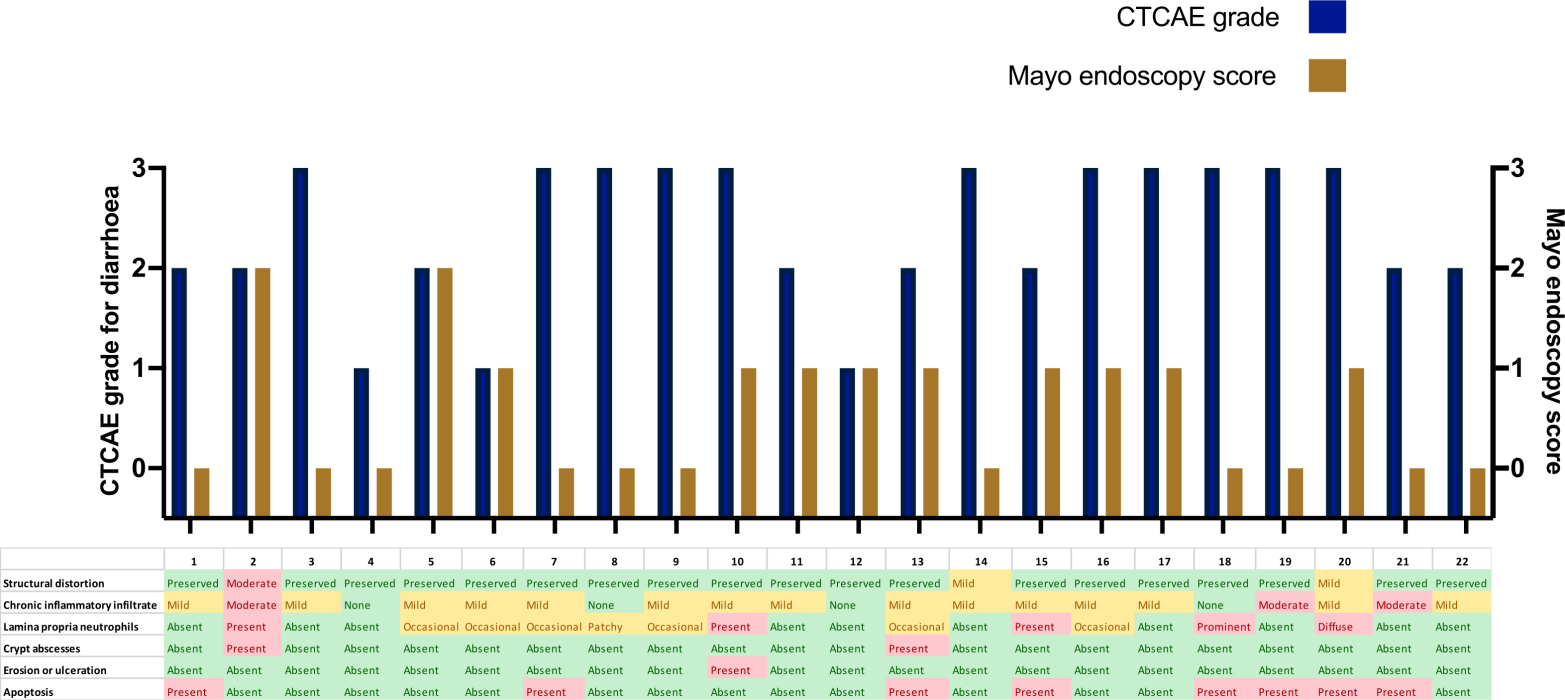
Clinical, endoscopic and histological characteristics of 22 patients. Severity of diarrhea (purple bars) according to the National Cancer Institute Common Terminology Criteria for Adverse Events (CTCAE), Version 5.0. CTCAE Grade 0: no symptoms of diarrhea. Grade 1: Increase of <4 stools per day over baseline. Grade 2: increase of 4–6 stools per day over baseline; limiting instrumental activities of daily living (ADL). Grade 3: increase of ≥7 stools per day over baseline; hospitalization indicated; limiting self-care ADL. Endscopic severity (brown bars) on baseline endoscopic assessment according to Mayo endoscopy score. Histological features shown in table format with green/yellow/red color scheme denoting severity of findings.

**Figure 2:**
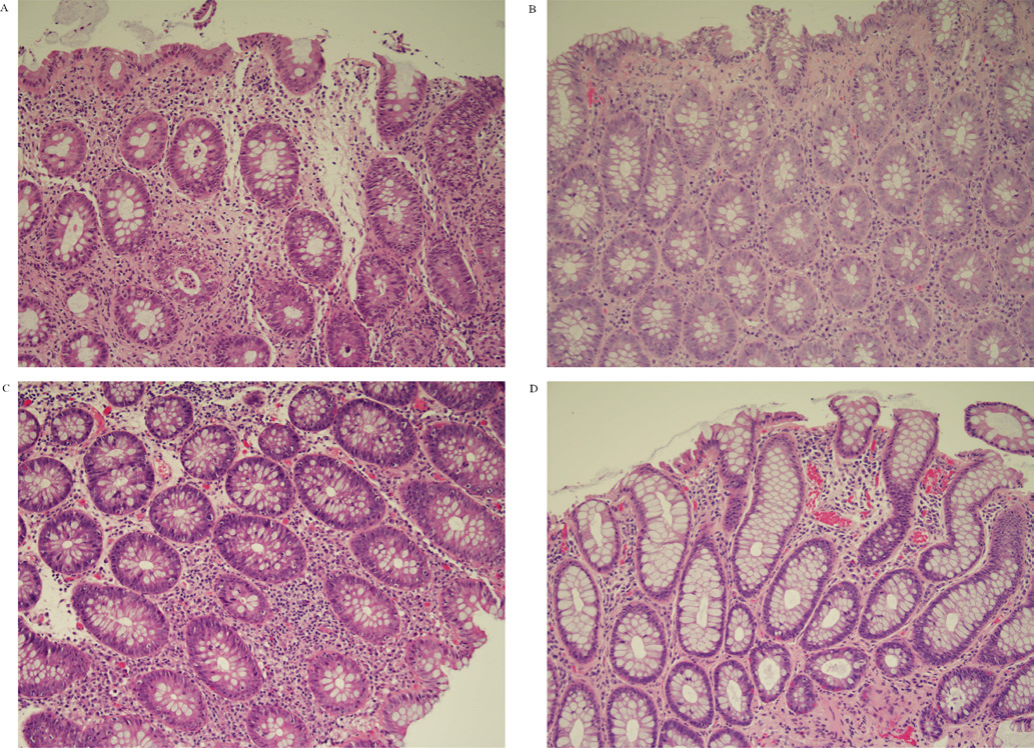
Histological images pretreatment and post-treatment with BD. (A) (Patient 2 pretreatment): increase in chronic inflammatory cells in the lamina propria and neutrophilic infiltrate. Crypt disruption and crypt abscesses. (B) (Patient 2 post-treatment): increase in plasma cells in lamina propria, apoptotic bodies in base of the crypts, no evidence of cryptitis or crpyt abscesses. (C) (Patient 7 pretreatment): congestion in the lamina propria and an increase in chronic inflammatory cells. Occasional apoptotic bodies are seen at the base of the crypt epithelium. (D) (Patient 7 post-treatment): normal large bowel mucosa. BD, beclomethasone dipropionate.

Clinical, endoscopic, and histological characteristics for each patient are shown in figure 1. Three patients had CTCAE grade 1 for diarrhea (Increase of<4 stools per day over baseline), eight had grade 2 (increase of 4–6 stools per day over baseline) and eleven had grade 3 (increase of>=7 stools per day over baseline). All patients underwent flexible sigmoidoscopy or colonoscopy at initial diagnosis. The median time between onset of symptoms and first endoscopy was 40 days (range 3–172 days). Eleven patients (50%) had no macroscopic features of inflammation (Mayo endoscopy score: 0). Nine patients had endoscopic features of mild colitis (granularity, decreased vascular pattern and/or erythema; Mayo endoscopy score: 1). Two patients had features of moderate colitis (marked erythema, erosions; Mayo endoscopy score: 2). One patient had an inflammatory stricture of the sigmoid colon. There was no correlation between CTCAE grade for diarrhea and Mayo endoscopy score (r=−0.30; p=0.18). Histological assessment of colonic biopsies demonstrated twenty patients (91%) had either mild or moderate inflammation (figure 1). Typical histological features included chronic inflammatory cell infiltrate (n=16; 73%), neutrophilic infiltrate (n=12; 55%) and apoptosis (n=8; 36%). Figure 2 shows histological images from two patients before and after treatment with BD.

### Clinical response to BD

**Figure 3:**
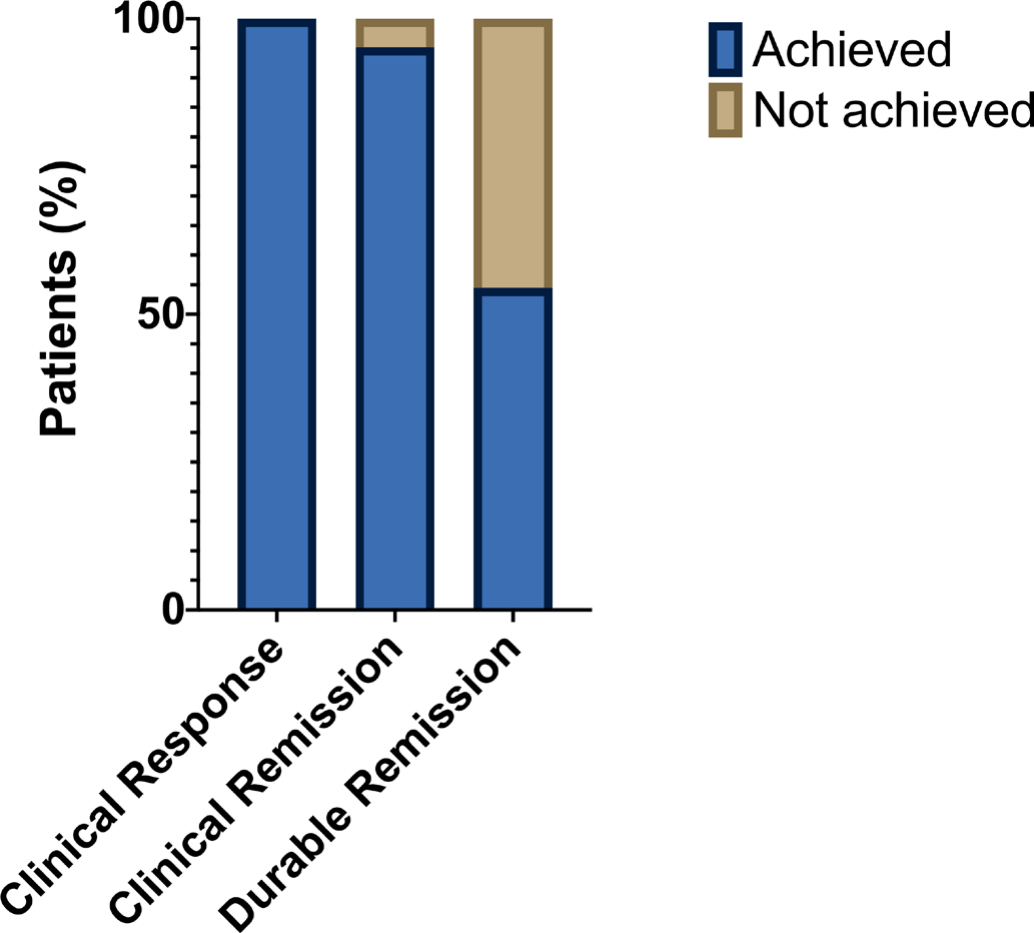
Clinical outcomes after a single course of BD. Shown are proportions of patients achieving a clinical response (left bar—defined as an improvement in diarrhea symptoms), clinical remission (middle bar—defined as a return to baseline stool frequency) and durable remission (right bar—defined as absence of relapse of diarrhea symptoms after treatment cessation). BD, beclomethasone dipropionate.

All 22 patients (100%) had a clinical response to BD (defined as a reduction in stool frequency of ≥ 1 loose stool per day). Time to clinical response ranged from 2 to 52 days. Twenty-one patients (95%) achieved clinical remission (defined as a return to baseline stool frequency) by completion of 28 days of BD (figure 3). All 13 patients who had been dependent on systemic corticosteroids were able to wean to a dose of prednisolone ≤ 5 mg once daily by the time of completing the course of BD (in such cases, to obviate the risk of Addisonian crisis, the minimum dose of prednisolone was set at 5 mg once daily pending the outcome of a Synacthen test to be performed after completion of BD). There were no reported adverse events in response to BD.

Twelve patients (55%) achieved durable remission after completion of BD, but 10 patients (45%) relapsed following completion of the 4 weeks course of treatment (figure 3). Seventy per cent of patients who relapsed had received combination CPI therapy, compared with 25% of patients with durable remission (table 2). Normal macroscopic appearanes at index endoscopy (Mayo endoscopy score 0) were seen in 70% of patients who relapsed, compared with 33% of patients with durable remission. There was no clear pattern of histological changes that differentiated patients who relapsed from those with durable remission.

**Table 2:** Characeteristics of relapsers and durable remitters

	Durable remitters (n=12)	Relapsers (n=10)
Immunotherapy		
Anti-PD-1 only	9	2
Combination therapy	3	7
Anti-CTLA-4/anti-PD-1 monotherapy or combination	0	1
Prior biologic therapy:		
Infliximab	4	9
Vedolizumab	2	1
Mayo endoscopy score		
0	4	7
1	7	2
2	1	1
Histology		
Structural distortion	2	1
Chronic inflammatory infiltrate	9	8
Lamina propria neutrophils	6	6
Crypt abscesses	2	0
Erosion or ulceration	0	1
Apoptosis	6	2

**Figure 4:**
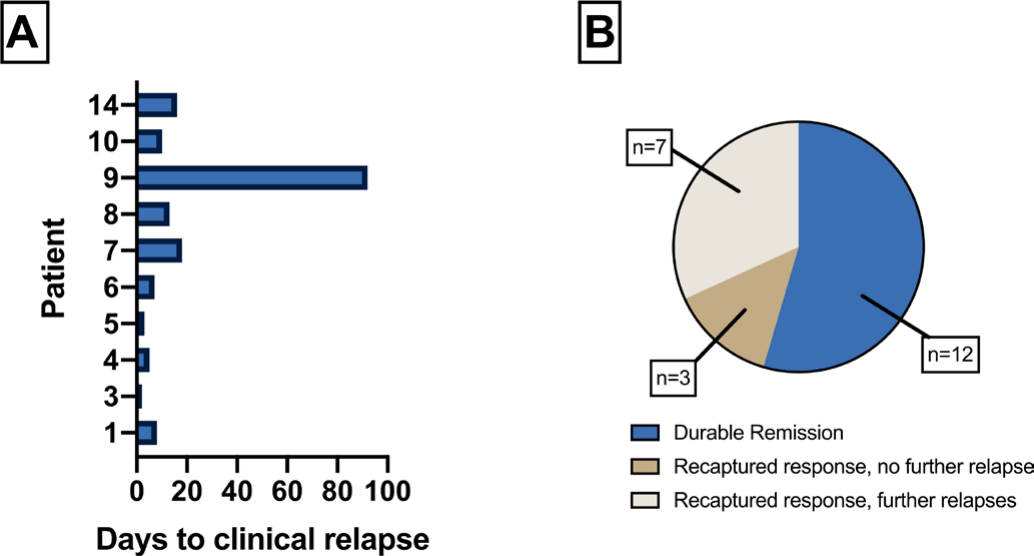
(A) Time course of clinical relapse after stopping BD in 10 patients who relapsed. (B) Final clinical outcomes following BD therapy. *In seven patients who had further relapses after >1 course of BD, four patients ultimately achieved remission without the need for alternative therapy. One patient was treated with 5-ASA, one patient was treated with MMF and one patient died following peritoneal malignant disease progression. BD, beclomethasone dipropionate; MMF, mycophenolate mofetil.

The median number of days to relapse was 9 (range 2–92) (figure 4A). The ultimate clinical outcomes are summarized in figure 4B. All 10 patients who relapsed were treated with a second course of BD and all recaptured response within several days. Seven patients had a further relapse after completing a second course of BD, of whom four recaptured response after subsequent rechallenge. Two relapsing patients failed to recapture response and were treated effectively with 5-ASA and mycophenolate mofetil (MMF), respectively. Four patients had multiple relapses requiring more than two courses of BD treatment. These patients remained on BD treatment intermittently for between five and eighteen months. Six months after BD treatment, one patient had malignant disease progression within the abdomen, with invasion of the descending colon by the primary renal tumor, resulting in perforation. A flexible sigmoidoscopy, which was performed 5 days before the diagnosis of perforation, showed mucosal edema only, with no ulceration. The perforation was not related to CPI-induced colitis or BD treatment. This patient received palliative care. Three patients (14%) restarted CPI therapy (two received anti-PD1 monotherapy and one received anti-CTLA-4 monotherapy). None of the patients developed relapse of colitis after restarting CPI.

## Discussion

Our study suggests that BD is safe and effective at inducing clinical remission in patients with CPI-induced colitis. All 22 patients had a reduction in diarrhea symptoms in response to treatment, and only 1 patient failed to achieve clinical remission after 28 days of treatment. Although the rate of symptomatic relapse was relatively high (45.5%) on cessation of BD, all patients recaptured response. Remarkably, in a group of patients with high rates of systemic corticosteroid dependence and infliximab treatment failure prior to BD treatment, only one patient needed subsequent re-institution of systemic immunosuppression with MMF to treat colitis symptoms.

The use of topical corticosteroids to treat CPI-induced colitis is not without precedent as there are retrospective data to suggest Budesonide may also be effective in this setting.^30 31^ To our knowledge, other than a prior case report from our group,^25^ this is the first analysis of BD use for the treatment of CPI-induced colitis. Although the current study reports on the efficacy of BD as a second line therapy in patients with refractory disease, its potential as a first line therapy in patients with mild or moderate (non-ulcerating) disease demands urgent investigation. Interestingly, a higher proportion of patients who relapsed after stopping BD had macroscopically normal index lower endoscopy (70%) than did those patients with durable remission (33%). Unsurprisingly, a higher proportion of the relapsing group had received combination immunotherapy (70% vs 25%). It is also notable that a significant minority of patients (32%) required more than two courses of BD therapy, effectively substituting systemic corticosteroid dependence for a period of topical therapy dependence. The requirement for multiple courses may reflect a particularly refractory phenotype, and future studies should set out to determine the optimal treatment period and predictors of relapse. We suggest that BD’s utility in CPI-induced GI inflammation is likely to be in patients with colonic involvement, as disintegration of enteric-coated BD has been shown to occur in the proximal colon in most individuals.^32^ Although active drug is released in the small bowel in more than a third of individuals,^32^ the distribution of small bowel involvement in CPI-induced enteritis is not well understood.

A key advantage of BD, is that its effectiveness permitted a rapid taper of concurrent systemic corticosteroids. Notably, the active metabolite of BD has >100 × higher binding affinity for the corticosteroid receptor than prednisolone.^33^ There are a number of potential benefits to expediting systemic corticosteroid wean, including reducing the burden of associated adverse side effects as well as removing the necessity for concurrent prophylactic medications such as protein pump inhibitors and antibiotics for *Pneumocystis jirovecii* pneumonia prophylaxis. The latter point is pertinent in light of emerging data which suggests that antibiotics and protein pump inhibitors incur an unfavorable impact on cancer outcomes in CPI treated patients via modulation of the gut microbiome.^34 35^ Finally, reducing the duration of systemic corticosteroids can also facilitate timely access to other anticancer therapy regimens.

In the era of COVID-19, the use of systemic immunosuppression has come into sharp focus because of concerns that such treatments increase the risk of infection and severe disease. COVID-19 in patients with cancer is associated with high mortality,^36^ and patients with cancer, as in our cohort, are often older (aged ≥60 years) with comorbidity, putting them at even greater risk. National guidelines have advised avoidance of systemic corticosteroids and consideration of topical corticosteroids where possible for flaring patients with IBD during the COVID-19 pandemic.^37^ The judicious use of topical corticosteroid therapy in patients with a less severe CPI-induced colitis phenotype has considerable appeal to reduce the risk of life-threatening infection.

We acknowledge several limitations to the current study. Data were collected from a retrospective review of electronic patient records, meaning that some information, including the exact timing of clinical response, was not available. Index endoscopic examinations were typically performed after the initiation of systemic corticosteroid therapy, meaning assessments might not fully reflect the disease phenotype at baseline. Although we observed a direct temporal relationship between BD treatment initiation and symptom improvement, which was recapitulated in those patients who relapsed and recaptured response, the absence of a study control arm means that we cannot be certain that BD was responsible. This patient cohort was notable for non-response or loss of response to systemic corticosteroids and, in those who received biologic therapy, non-response to full induction with infliximab and vedolizumab. Data from a recent study suggest that over 80% of patients achieving corticosteroid-free clinical remission with infliximab will respond to the drug within 7 days,^17^ whereas the patients in the current study were deemed to have had an inadequate reponse, leading to commencement of BD. Nonetheless, in most cases there was no wash-out period between prior biologic treatments and initiation of BD, meaning it is conceivable that some clinical benefit might be attributable to other treatments than BD. Finally, in the majority of patients, post-treatment endoscopic and histological data were not available, precluding the use of these harder endpoints as markers of treatment success.

## Conclusions

Our data suggest that BD is a safe and effective anti-inflammatory agent, in the treatment of mild to moderate or microscopic CPI-induced colitis, which is refractory to systemic corticosteroids. These results position BD as an attractive therapeutic strategy in the management of this emerging mucosal disease. Further randomized controlled studies are needed to establish its role in the treatment algorithm.

## Data Availability

Data are available on reasonable request. The datasets used and analyzed during the current study are available from the corresponding author on reasonable request.
